# Modern Dressings in Prevention and Therapy of Acute and Chronic Radiation Dermatitis—A Literature Review

**DOI:** 10.3390/pharmaceutics14061204

**Published:** 2022-06-06

**Authors:** Konrad Zasadziński, Mateusz Jacek Spałek, Piotr Rutkowski

**Affiliations:** 1 Department of Radiotherapy I, Maria Sklodowska-Curie National Research Institute of Oncology, 02-781 Warsaw, Poland; konrad.zasadzinski@pib-nio.pl; 2 Department of Soft Tissue/Bone Sarcoma and Melanoma, Maria Sklodowska-Curie National Research Institute of Oncology, 02-781 Warsaw, Poland; piotr.rutkowski@pib-nio.pl

**Keywords:** dermatitis, radiotherapy, biological dressings, bandages, necrosis, ulcer, radiodermatitis, radiation-protective agents, radiation injuries

## Abstract

Radiotherapy is an integral part of modern oncology, applied to more than half of all patients diagnosed with cancer. It can be used alone or in combination with surgery or chemotherapy. However, despite the high precision of radiation delivery, irradiation may affect surrounding healthy tissues leading to the development of toxicity. The most common and clinically significant toxicity of radiotherapy is acute and chronic radiation dermatitis, which could result in desquamation, wounds, nonhealing ulcers, and radionecrosis. Moreover, preoperative radiotherapy impairs wound healing after surgery and may lead to severe wound complications. In this review, we comprehensively discuss available types of dressings used in the management of acute and chronic radiation dermatitis and address their efficacy. The most effective ways of preventing acute radiation dermatitis are film dressings, whereas foam dressings were found effective in its treatment. Data regarding dressings in chronic radiation dermatitis are scarce. This manuscript also contains authors’ consensus.

## 1. Introduction

Radiation-induced dermatitis (RID) is the most frequent side effect of radiotherapy and affects approximately 90–95% of patients exposed to therapeutic radiation, with 87% of patients experiencing moderate-to-severe skin reactions [1]. Acute radiation dermatitis (ARD) usually occurs within 90 days of exposure to ionizing radiation, whereas chronic radiation dermatitis (CRD) may develop many years after the completion of treatment. Both ARD and CRD are associated with radiation exposure of 2–50 Gy [2,3]. ARD occurs mostly in particular sites, namely, the neck, face, extremities, chest, and abdomen [4]. The main symptoms of ARD include erythema, dry and wet desquamations, and ulceration [5]. The manifestation of CRD is more complex and covers events from mild fibrosis to secondary cancers [3].

Modern radiation techniques such as intensity-modulated radiotherapy (IMRT) and computed-tomography-based planning algorithms enabled a significant reduction in the rates of severe RID, mostly due to sparing of the skin and subcutaneous tissue [6]. Nevertheless, moderate and severe forms of RID can still result in serious impairments of patients’ quality of life and may also be a major cause of nonadherence or treatment interruptions [7]. Hence, appropriate and efficient management of RID plays a crucial role in radiation oncology supportive care.

Radiation-associated skin toxicity is complex and dependent on a variety of factors, such as total dose delivered, fractionation regimen, and volume of irradiated tissue, as well as concomitant systemic therapy and comorbidities. Therapeutic radiation exhibits biological effects within hours to weeks after exposure, causing extensive genetic damage irreversibly breaking double strands in nuclear and mitochondrial DNA, and inhibiting cells’ ability to divide and replicate. This damage, along with other structural tissue destruction, generation of reactive oxygen species, a decrease in the functional stem cell population, initiation of epidermal and dermal inflammatory responses, and skin cell necrosis, results in RID [8].

To date, no gold standards in the management of ARD and CRD have been established. Although numerous topical and systemic medications are available for treatment and prevention of radiation-associated skin reactions, conclusions of various clinical trials often contradict each other and lack universality, mostly due to the lack of high-quality and large-sample studies. Therefore, the management of RID is often empirical and commonly based on personal experience supported by weak scientific evidence [9,10].

Although the standard forms of RID management were previously based on topical agents, mostly aqueous or steroid creams, recently more attention has been turned towards various forms of dressings applied either as RID prophylaxis (from the beginning of radiation therapy) or as a treatment form (applied at the onset of skin damage signs). Their potential advantage over topical creams is their common feature of creating a stable moist environment that enables faster re-epithelialization of radiation-damaged skin. Some dressings also possess antimicrobial and anti-inflammatory features that additionally facilitate radiation damage prevention or healing [7,11].

The objective of this narrative review is to discuss various types of dressings previously studied for the treatment and prevention of RID and show options that have proved successful, resulting in satisfactory clinical outcomes. To clearly present this complex issue, we divided the text into the following five sections: description of the main types of dressings, dressings in ARD and CRD, novel technologies and summary with recommendations. The issues of wound healing complications after surgery with prior radiotherapy and vacuum-assisted closure therapy were out of the scope of this narrative review.

## 2. Types of Dressings and Wound Management

The development of surgery and various dressings subtypes enabled effective wound control. The simplest division covers two groups, non-absorbing (like films) and absorbing (such as foams). The introduction of additional materials or substances allow modification of wound microenvironment, namely exudate, microflora, epithelization, healing and scar formation [12]. The indication for a particular dressing may be based on the acronym T.I.M.E. that describes the general rules of wound preparation. That covers four domains, i.e., tissue management, inflammation and infection control, moisture balance, and epithelial edge advancement [13,14]. The proposed T.I.M.E. for RID was proposed in Supplementary Table S1.

However, the complexity and unpredictability of RID development and different pathophysiology compared to in the case of other wounds limit the easy application of T.I.M.E. into routine practice in radiation oncology. First, the traumatic factor, ionizing radiation, constantly affects tissues for several days. Second, all phenomena may occur at the same time, because various areas of the skin and subcutaneous tissue might receive different doses. Third, microflora of the skin may be also affected by radiation, immunosuppression, and concomitant systemic therapy. Moreover, natural reepithelization usually begins within ten days [15]. Finally, RID-related wound care should consider the possible effect of applied treatment (like thick foam dressing) to the skin that may lead to changes in dose distribution.

## 3. Acute Radiation Dermatitis

### 3.1. Definition and Classification

**Figure 1:**
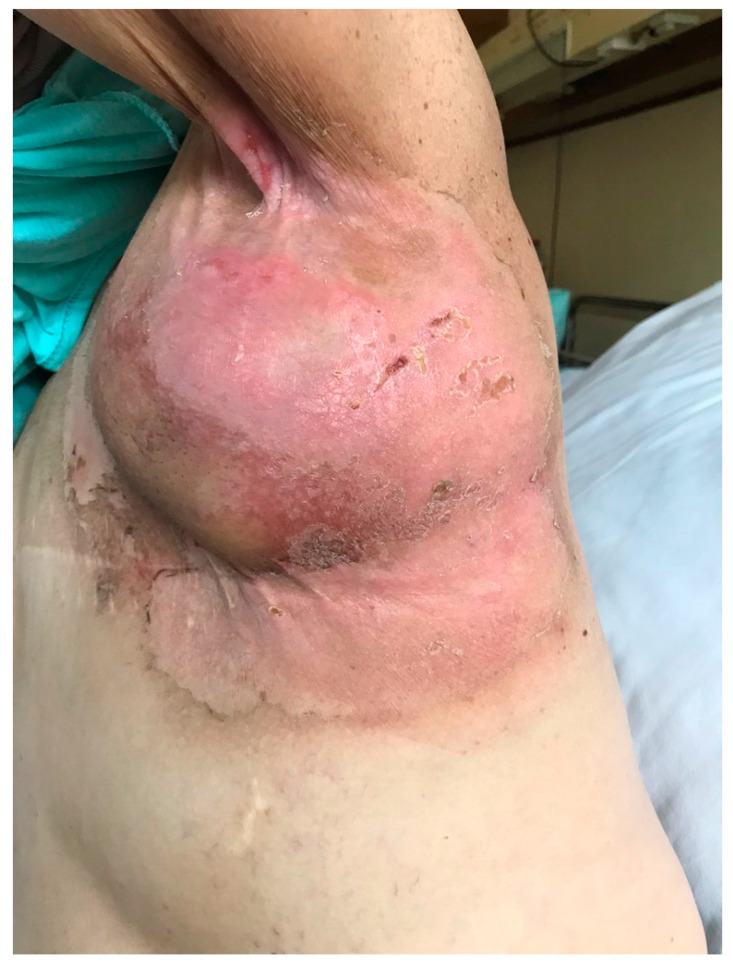
Grade 2 acute radiation dermatitis.

ARD may present in the form of erythema, dry and moist desquamation, skin necrosis, ulcers, as well as bleeding. The Radiation Therapy Oncology Group (RTOG) and the European Organization for Research and Treatment of Cancer (EORTC) have developed a standardized grading system to evaluate acute radiation-induced skin toxicity (Table 1) [16]. A measurement tool has also been created that helps evaluate ARD using both patient symptoms and healthcare professionals’ assessment scales. The tool is called the Radiation-Induced Skin Reaction Assessment Scale (RISRAS) [17]. Another important classification of ARD more frequently used in clinical trials than RTOG/EORTC scale is Common Terminology Criteria for Adverse Events (CTCAE) version 5.0 (Table 1). Figure 1 presents an example of grade 2 acute radiation dermatitis.

### 3.2. Dressings in ARD Prevention and Treatment

Several dressings were investigated in both ARD prophylaxis and treatment. The main aim of their application is the reduction of ARD severity or severity of related symptoms (such as pain) and subsequent improvement of treatment tolerance. Our review focuses on available data of significant scientific evidence obtained from randomized controlled trials and cohort studies. The summary of discussed literature data regarding ARD prevention and treatment is presented in Supplementary Table S2.

#### 3.2.1. Film and Membrane Dressings

Mepitel Film is a semi-permeable dressing/film based on Safetac technology. The film can be used prophylactically, starting from the first day of radiotherapy, and the transparency grants skin appearance assessment without the need for dressing removal [18]. Five randomized trials were conducted that assessed the potential reduction of ARD rates with Mepitel Film used as ARD prophylaxis. A Danish trial by Møller and colleagues involved 101 patients treated for breast cancer who were randomized to cover either the lateral or medial part of their chest with Mepitel film [19]. The primary endpoint was patient-reported symptoms and experience. A secondary endpoint was radiotherapy staff evaluation of dermatitis using the RTOG scale. Out of 79 patients included in the final statistical analysis, a statistically significant proportion reported a lower level of pain, itching, burning sensation as well as a reduced sensitivity within the area covered by Mepitel film. There was no statistical difference in staff-evaluated RID rates on the last day of radiotherapy among the whole study group. In selected groups of patients, i.e., post-mastectomy patients and patients treated with a total dose of 50 Gy, there was a significant difference in ARD rates. However, fourteen days after radiotherapy, the difference in ARD rates was overall non-significant in all groups of patients. The original randomized trial by Herst and colleagues provided evidence of Mepitel film effectiveness in reducing the severity of skin reactions in breast cancer patients [20]. A total of 78 participants contributed data for analysis. Lateral and medial halves of the skin areas to be irradiated were randomized to Mepitel Film or aqueous cream. Skin reaction severity was assessed using RISRAS and RTOG scales. Overall skin reaction severity (RISRAS) was reduced by 92% in favor of Mepitel Film. All patients developed some form of reaction in cream-treated skin, which progressed to moist desquamation in 26% of patients. Only 44% of patients had a skin reaction under the Mepitel Film, which did not progress to moist desquamation in any of the patients. Three other RCTs with relatively smaller sample groups have also been conducted that tested prophylactic use of Mepitel film in head and neck cancer patients. One of them did not reach its primary endpoint due to a limited tolerance of Mepitel film [21]. Two other studies demonstrated reduced risks of developing ARD and a decrease in ARD severity in the study groups [22,23]. The efficacy of Mepitel film in ARD prophylaxis was also demonstrated in a single-arm feasibility study by Yee and colleagues involving 30 patients irradiated for breast and chest wall neoplasms. Complete prevention of RTOG grade 3 ARD and a significant reduction in grade 2 cases were obtained. Moist desquamation, however, could not be completely prevented [24]. A retrospective review carried out by Oshin et al. showed significantly lower rates of moist desquamation after prophylactic use of Mepitel film in patients with breast cancer [25].

Two trials verifying the use of polymeric membrane dressings for ARD treatment in head and neck cancer patients were conducted. A randomized controlled trial by Scott presented mixed outcomes, demonstrating a significant reduction of self-reported pain and improved quality of life with no effect on ARD healing rates [26]. Another study, a single-arm controlled trial by Hegarty and Wong, confirmed polymeric membrane dressings’ superiority over standard care in ARD treatment [27]. Another type of polymeric dressing was also tested for ARD prevention. Two studies conducted by Schmeel et al. investigated the use of Hydrofilm (polyurethane film) dressing in ARD prophylaxis in breast cancer patients. A 2018 randomized controlled trial demonstrated a significant reduction in ARD rates in the study group [28]. Moreover, complete prevention of moist desquamation was also obtained. In a 2019 self-controlled trial, the authors confirmed these findings [29]. The study proved a reduction of staff-assessed dermatitis signs as well as complete prevention of moist desquamation. Furthermore, patient-reported symptoms such as itching, burning, and pain were also significantly diminished in the study group.

A large-sample Japanese study showed a beneficial effect of a thin-film dressing application for ARD prevention in patients undergoing proton beam therapy for prostate cancer [30].

Soft silicone film dressing was recently tested in a randomized controlled trial by Zou and colleagues [31]. The study involved 100 patients treated for various cancer types. The experimental group that received soft polysiloxane film for ARD prophylaxis presented a significantly lower incidence of dermatitis signs than the control group. Also, the healing of ARD lasted shorter in the experimental group.

#### 3.2.2. Foam Dressings

Mepilex Lite (Molnlycke Health Care, Gothenburg, Sweden) is a thin dressing composed of an outer polyurethane film, an absorbent layer, and a soft silicone wound contact layer that enables adherence to wounds with low-to-medium exudate levels. It can be left in place for up to 14 days, whereas the secondary dressing can be changed as frequently as required to avoid irritation of the wound bed [32]. The efficacy of Mepilex Lite in RID management was studied in a clinical trial conducted by Zhong and colleagues who assessed a group of 88 head and neck cancer patients developing ARD during radiotherapy [33]. The study sample of 43 patients who received Mepilex Lite dressings showed significantly faster healing times than the control group (median healing time was 16 vs. 23 days, respectively; *p* = 0.009). Other parameters impacting patients’ quality of life were also assessed, among which patients’ sleep was significantly improved in the study group. Diggelmann and colleagues studied 24 patients irradiated for breast cancer who developed ARD [34]. Each of the erythematous areas (*n* = 34) was randomly divided into two groups; the first group was treated with Mepilex Lite dressing and the other with standard care, i.e., an aqueous cream. There was a significant reduction in the severity of acute radiation dermatitis in the areas in which Mepilex Lite dressings were applied compared with the control areas. Paterson et al. also confirmed Mepilex Lite efficacy in ARD management [35]. Although the incidence rates of moist desquamation were equal for the study and control (aqueous cream) groups, their trial showed a significant reduction in overall skin reactions severity by 41% (*p* < 0.001), along with a reduction in the average moist desquamation score by 49% (*p* = 0.043) in favor of Mepilex-covered skin areas.

#### 3.2.3. Gel Dressings

Hydrogel and hydrocolloid dressings have been used in treating moderate and severe forms of ARD that involve moist desquamation. They facilitate the maintenance of a wet environment over de-epithelialized skin, and thus are considered to accelerate healing.

Three randomized controlled trials investigated hydrogel dressings for treating moist desquamation, the first compared hydrogel to a gentian violet dressing in patients with breast or head and neck cancers [36], the second compared hydrogel to a simple dry dressing for people with ARD in the head and neck, breast and anorectal regions [37], and the third and largest study compared Hydrosorb^®^ (hydrogel dressing without oil components) to a water-based spray in patients treated for breast cancer [38]. The only study that confirmed Hydrogel’s efficacy was the Gollins et al. trial, which showed hydrogel dressing superiority to a gentian violet dressing in terms of healing rates in patients with moist desquamation [36]. Two other trials, with significantly larger study groups, could not confirm these findings, with one study showing even prolonged healing times in the Hydrogel group compared to standard care, i.e., dry dressing.

StrataXRT is a silicone-based film-forming, self-drying, semi-occlusive, non-resorbable, topical gel preparation, consisting of polydimethylsiloxanes, siloxanes, and alkyl methyl silicones. The dressing is designed to promote a moist wound-healing environment. When applied topically, StrataXRT dries to form a thin, flexible, protective layer that is gas permeable and waterproof. This environment leads to rapid wound healing and faster skin recovery [39]. In a relatively large single-blind randomized controlled trial by Chan et al., a reduced risk of developing ARD was obtained with prophylactic application of StrataXRT dressings [40]. The study involved 197 head and neck cancer patients randomized to receive either standard care (Sorbolene cream) or StrataXRT dressing from the beginning of radiation therapy. There was a significantly lower incidence of RTOG grade 2 and 3 ARD in the study group. Also, delayed development of skin toxicity was demonstrated in the dressing group. There was no difference in patient-reported symptoms. Two other studies verifying the prophylactic use of StrataXRT confirmed these findings. An RCT involving 56 patients treated for breast cancer demonstrated a reduction in objectively measured ARD severity in the StrataXRT group [39]. The other study proved StrataXRT’s noninferiority to Mepitel film in ARD prevention [41]. The only trial studying the therapeutic use of StrataXRT for ARD was a prospective study by Quilis et al., which showed a significant improvement in RISRAS score for patients receiving StrataXRT dressings at the onset of dermatitis signs [42].

Application of 3M Cavilon Barrier Film was tested as a form of ARD prevention. Three randomized controlled studies were carried out that involved patients treated for breast cancer. Only one of them demonstrated the superiority of this dressing over sorbolene cream in reducing the rates of moist desquamation and pruritus [43]. Two newer randomized controlled trials did not confirm these findings [44,45].

#### 3.2.4. Silver-Containing Dressings

Silver nylon dressings are nonadhesive nanocrystalline silver-coated material, used clinically as a burn dressing with satisfactory outcomes resulting from its antimicrobial activity against Gram-positive and Gram-negative bacteria as well as some fungal infections [46]. Aquino-Parsons et al. studied 196 patients treated with whole-breast radiation therapy [47]. They showed that there was no benefit of silver-leaf nylon dressings for the prevention of acute grade 3 ARD compared with patients who received standard skin care. However, the incidence of itching in the last week of radiation and one week post-treatment was lower among the patients who used the dressings. The Niazi et al. study compared prophylactic use of silver clear nylon dressing vs. no prophylaxis with sulfadiazine cream applied at the onset of RID [48]. The trial demonstrated a significant reduction in the severity of RID in the study group on the last day of treatment. However, the skin reaction assessed two weeks after treatment completion showed no statistically significant difference between the study and control groups. Prophylactic use of silver dressings also proved to be effective in a single-arm controlled trial by Vuong et al., which covered 30 patients treated for gynecological and anal cancers [49]. The mean dermatitis score was significantly lower in patients who used dressings for RID prevention in comparison with a historical control group. A self-controlled trial by Vavassis et al., which studied silver nylon dressings for the treatment of ARD in patients with head and neck cancers, showed no superiority of these dressings over the standard care in terms of RID severity measured using the RTOG scale [50]. There was, however, a significant improvement in pain control in favor of silver dressing.

#### 3.2.5. Biodressings

Biodressings are highly advanced biomaterials that combine conventional fibers with bioactive molecules such as growth factors or stem cells [51]. The main aim of their use is to speed up the healing process. Such biomaterials were used in the management of severe ARD. In a small prospective observational study, Lee and colleagues demonstrated a significant acceleration in the healing of severe ARD in patients irradiated for head and neck cancers treated with a foam dressing containing epidermal growth factor [52]. A dressing in the form of a gauze impregnated with granulocyte-macrophage colony-stimulating factor tested by Kouvaris et al. showed to be efficacious in the prevention and treatment of ARD in women undergoing radiotherapy for vulvar cancer [53].

The use of lyophilized and irradiated human amniotic membrane as a biological dressing for grade 2 and 3 ARD treatment was evaluated in an Indian study by Lobo-Gajiwala and Sharma [54]. The authors used such a biodressing in fourteen patients who developed moist desquamation in groin folds and natal cleft after pelvic radiotherapy. They observed rapid healing of ARD in all treated patients, achieving a median of seven days after dressing application.

#### 3.2.6. Other Dressings

A retrospective cohort analysis by Bonomo et al. showed an improvement in treatment tolerability in head and neck cancer patients undergoing radiotherapy with concurrent cetuximab who received calcium alginate dressings for the therapy of severe ARD involving moist desquamation [55]. In another analysis, the dry non-adherent absorbent dressing was shown to be ineffective in ARD treatment in patients with head and neck cancers [56].

## 4. Chronic Radiation Dermatitis

### 4.1. Definition and Classification

**Figure 2:**
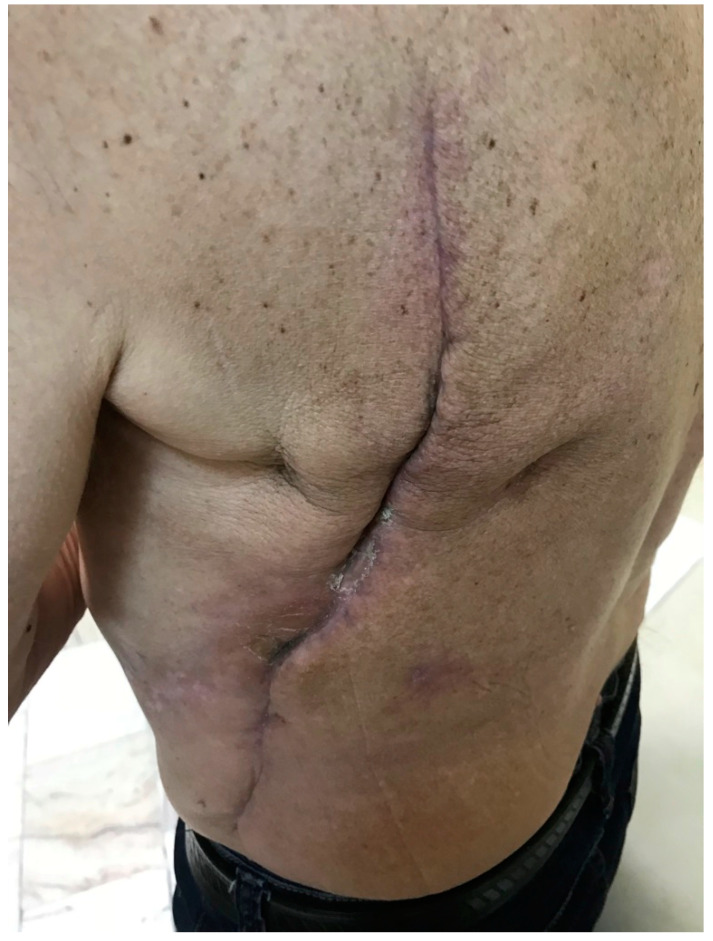
Grade 1 chronic radiation dermatitis.

CRD is an irreversible and progressive complication of radiotherapy, which usually influences patients’ quality of life. It can occur suddenly even several years after irradiation. CRD covers chronic ulcers including necrosis, radiation-induced keratosis, telangiectasias, fibrosis, as well as secondary skin cancers [3]. The RTOG/EORTC classification of CRD was shown in Table 2. Figure 2 presents an example of grade 1 skin fibrosis with anthropic changes.

CTCAE classification does not directly mention the term CRD but describes its several related manifestations, namely, all events related to late fibrosis, ulceration, and necrosis.

### 4.2. Dressings in CRD Prevention and Treatment

Whereas radiation-induced fibrosis is mostly managed with other methods, the most frequent indications for dressings in CRD are chronic ulceration and necrosis. Unfortunately, the available data are scarce. There are no randomized nor single-arm prospective trials regarding the efficacy of modern dressings in the management of severe CRD. Thus, we did not prepare a similar summary of evidence as in the case of ARD. However, several authors reported such attempts in retrospective analyses and case reports. The most important and promising dressings for ulcers and necrosis related to CRD are biodressings. Below, we have described and discussed biodressings that were investigated in humans.

American researchers reported a case of a patient with chronic radiation necrosis treated with a lyopreserved placental membrane containing viable cells [57]. The 73-year-old woman received postoperative radiotherapy due to squamous cell carcinoma of the right medial ankle, and she developed a chronic necrotic wound refractory to conventional treatment (collagen dressings, honey-impregnated dressings, topical and oral antibiotics). It was decided to use vLPM (GrafixPL PRIME^®^; Osiris Therapeutics, Inc., Columbia, MD, USA). According to the description, this material contains a lyopreserved placental tissue allograft that retains the extracellular matrix, growth factors, and endogenous neonatal mesenchymal stem cells, fibroblasts, and epithelial cells of the native tissue. The dressing provided the desired effect (re-epithelialization and wound closure) after 98 days without any significant adverse events.

Another case report described a 59-year-old woman with radiation necrosis on the trunk which developed after total body electron irradiation with a relatively low dose of 36 Gy for cutaneous T-cell lymphoma [58]. CRD was confirmed by biopsy. The wound was treated with PDGF BB 0.01 gel (Regranex^®^ 0.01 gelanssen-Cilag, Neuss, Germany), covered by a hydrophilic copolymer membrane (Omiderm^®^; Omikron Scientific Ltd., Rehovot, Israel, distributed by Idel Medical Service, Hamburg, Germany). The authors reported re-epithelialization and granulation as well as pain reduction.

A group of Italian researchers obtained excellent results in the treatment of 20 patients with severe CRD with autologous adipose-derived stem cells therapy, including patients with ulcers, necrosis, fibrosis, telangiectasia, atrophy, and retraction [59]. A significant clinical improvement was observed in all but one patient.

Recombinant human platelet-derived growth factor-BB (rhPDGF) gel was found effective in another 47-year-old patient who suffered from chronic radiation-induced ulceration of the neck area that persisted for 12 years [60]. He underwent radiochemotherapy for locally advanced nasopharyngeal carcinoma. Six months of RhPDGF gel application enabled satisfactory granulation to apply a split-thickness skin graft.

## 5. Novel Technologies

Recent developments in modern dressings used in cancer patients are focused on two main groups, namely, interactive dressings and biodressings. The first group includes various substances that affect wound microenvironment. Intensively studied and described examples are hyaluronic acid, alginate, and collagen-coated dressings [61]. However, none of them was proven effective in RID prevention or treatment.

Biodressings constitute the most promising group of modern dressings that may have a significant role in RID prevention and treatment in the future. Biodressings provide the gradual release of the integrated biomolecules that enable faster healing. An interesting and promising approach is the use of functional hydrogels that present complex properties ensuring proper wound healing [62,63]. Especially interesting are the multifunctional photoresponsive hydrogels that combine pro-healing effect of light-based therapy with properties of hydrogels. Other studies also describe the various hydrogels integrated with biopolymers that may accelerate healing of post-radiation wounds [64,65,66,67,68]. Several biodressing groups were studied and widely described in the literature [61,69,70,71,72]. However, this group is rarely investigated in patients who undergo radiotherapy. The reason could be the risk of decrease of lethal damage caused by ionizing radiation to cellular DNA. Photon radiotherapy acts mostly through free radicals. Thus, antioxidative properties of the biodressings may affect efficacy of radiotherapy [73]. As a result, this group of dressings should be used with caution in patients with ARD.

## 6. Summary and Recommendations

**Figure 3:**
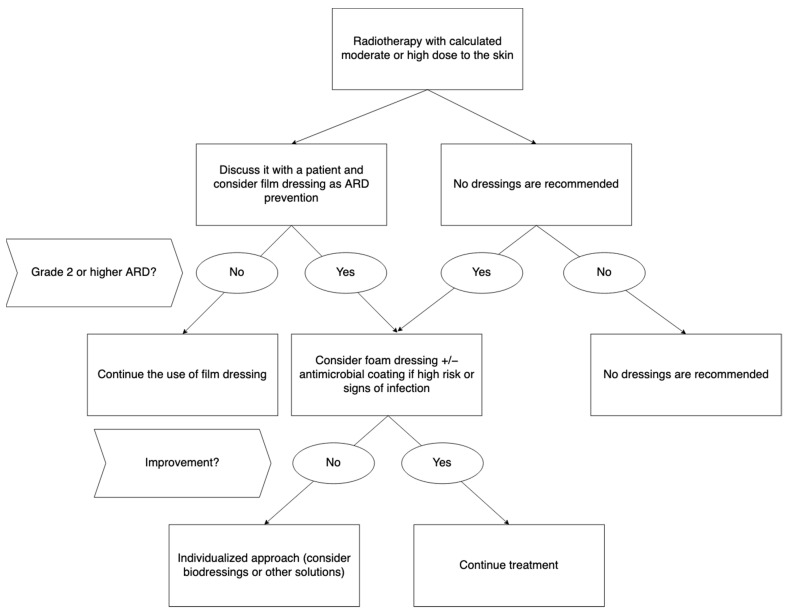
Recommended dressings for prevention and treatment of acute radiation dermatitis. Abbreviations: ARD—acute radiation dermatitis.

Available data suggest that the use of dressings as a preventive measure in RID management may be both efficacious and cost-effective, reducing skin-related complications and preventing treatment interruptions. Based on available evidence, film dressings could be a valuable prevention method against ARD. Foam dressings may be used for the treatment of severe ARD, whereas hydrogel dressings seem to be ineffective in that indication. Figure 3 presents authors’ consensus and recommendation regarding prevention and treatment of ARD.

Nevertheless, due to the high heterogeneity of RID manifestation, some patients require an individualized approach. Moreover, due to the lack of additional analyses (real clinical benefit measured in oncological outcomes, cost-effectiveness), any of the discussed dressings cannot be recommended as a part of routine clinical practice.

Evidence-based data on the use of any dressings in ulceration and necrosis as a manifestation of CRD are scarce. Biodressings are a promising group of modern dressing that showed preliminary efficacy in both ARD and CRD. However, further investigations of their potential are warranted.

## Figures and Tables

**Table 1:** The classification of acute radiation dermatitis.

Grade	RTOG/EORTC	CTCAE 5.0
0	No change over baseline	NA
1	Follicular, faint or dull erythema/epilation/dry desquamation/decreased sweating	Faint erythema or dry desquamation
2a	Tender or bright erythema +/− dry desquamation	Moderate-to-brisk erythema; patchy moist desquamation, mostly confined to skin folds and creases; moderate edema
2b	Patchy moist desquamation, moderate edema	
3	Confluent, moist desquamation other than skin folds, pitting edema	Moist desquamation in areas other than skin folds and creases; bleeding induced by minor trauma or abrasion
4	Ulceration, hemorrhage, necrosis	Life-threatening consequences; skin necrosisor ulceration of full-thickness dermis; spontaneous bleeding from the involved site; skin graft indicated
5	NA	Death

**Abbreviations:** CTCAE 5.0—Common Terminology Criteria for Adverse Events version 5.0; NA—not applicable; RTOG/EORTC—the Radiation Therapy Oncology Group/the European Organization for Research and Treatment of Cancer (EORTC).

**Table 2:** The classification of chronic radiation dermatitis according to the Radiation Therapy Oncology Group/the European Organization for Research and Treatment of Cancer (EORTC).

Grade	RTOG/EORTC
0	No change over baseline
1	Slight atrophy; pigmentation change; some hair loss
2	Patch atrophy; moderate telangiectasia; total hair loss
3	Marked atrophy; gross telangiectasia
4	Ulceration

**Abbreviations:** RTOG/EORTC—the Radiation Therapy Oncology Group/the European Organization for Research and Treatment of Cancer (EORTC).

## Data Availability

Not applicable.

## Supplementary Materials

The following supporting information can be downloaded at: https://www.mdpi.com/article/10.3390/pharmaceutics14061204/s1, Table S1: Proposed T.I.M.E. wound bed preparation for acute and chronic radiation dermatitis, Table S2: Dressings for prevention and treatment of acute radiation dermatitis [19,20,21,22,23,24,25,26,27,28,29,30,31,33,34,35,36,37,38,39,40,42,43,44,45,47,48,49,50,52,53,54,55,56,74].

## References

[1] Chan R.J., Webster J., Chung B., Marquart L., Ahmed M., Garantziotis S. (2014). Prevention and Treatment of Acute Radiation-Induced Skin Reactions: A Systematic Review and Meta-Analysis of Randomized Controlled Trials. BMC Cancer.

[2] Bray F.N., Simmons B.J., Wolfson A.H., Nouri K. (2016). Acute and Chronic Cutaneous Reactions to Ionizing Radiation Therapy. Dermatol. Ther..

[3] Spałek M. (2016). Chronic Radiation-Induced Dermatitis: Challenges and Solutions. Clin. Cosmet. Investig. Dermatol..

[4] Brown K.R., Rzucidlo E. (2011). Acute and Chronic Radiation Injury. J. Vasc. Surg..

[5] Kiprian D., Szykut-Badaczewska A., Gradzińska A., Czuwara J., Rudnicka L. (2022). How to Manage Radiation-Induced Dermatitis?. Nowotw. J. Oncol..

[6] Pignol J.-P., Olivotto I., Rakovitch E., Gardner S., Sixel K., Beckham W., Vu T.T.T., Truong P., Ackerman I., Paszat L. (2008). A Multicenter Randomized Trial of Breast Intensity-Modulated Radiation Therapy to Reduce Acute Radiation Dermatitis. J. Clin. Oncol..

[7] Rosenthal A., Israilevich R., Moy R. (2019). Management of Acute Radiation Dermatitis: A Review of the Literature and Proposal for Treatment Algorithm. J. Am. Acad. Dermatol..

[8] Fowble B., Yom S.S., Yuen F., Arron S. (2016). Skin Care in Radiation Oncology: A Practical Guide.

[9] Bolderston A., Lloyd N.S., Wong R.K.S., Holden L., Robb-Blenderman L., Supportive Care Guidelines Group of Cancer Care Ontario Program in Evidence-Based Care (2006). The Prevention and Management of Acute Skin Reactions Related to Radiation Therapy: A Systematic Review and Practice Guideline. Support. Care Cancer.

[10] Hegedus F., Mathew L.M., Schwartz R.A. (2017). Radiation Dermatitis: An Overview. Int. J. Dermatol..

[11] Singh M., Alavi A., Wong R., Akita S. (2016). Radiodermatitis: A Review of Our Current Understanding. Am. J. Clin. Dermatol..

[12] A Practical Guide to the Most Commonly Used Dressings in Wound Care. https://www.thepmfajournal.com/error-page/a-practical-guide-to-the-most-commonly-used-dressings-in-wound-care.

[13] Halim A.S., Khoo T.L., Saad A.Z.M. (2012). Wound Bed Preparation from a Clinical Perspective. Indian J. Plast. Surg..

[14] Wound Bed Preparation in Practice EWMA Position Document—Wounds International. https://www.woundsinternational.com/resources/details/wound-bed-preparation-practice-ewma-position-document.

[15] Mendelsohn F.A., Divino C.M., Reis E.D., Kerstein M.D. (2002). Wound Care after Radiation Therapy. Adv. Skin Wound Care.

[16] Cox J.D., Stetz J., Pajak T.F. (1995). Toxicity Criteria of the Radiation Therapy Oncology Group (RTOG) and the European Organization for Research and Treatment of Cancer (EORTC). Int. J. Radiat. Oncol. Biol. Phys..

[17] Noble-Adams R. (1999). Radiation-Induced Skin Reactions. 2: Development of a Measurement Tool. Br. J. Nurs..

[18] Fernández-Castro M., Martín-Gil B., Peña-García I., López-Vallecillo M., García-Puig M.E. (2017). Effectiveness of Semi-Permeable Dressings to Treat Radiation-Induced Skin Reactions. A Systematic Review. Eur. J. Cancer Care.

[19] Møller P.K., Olling K., Berg M., Habæk I., Haislund B., Iversen A.-M., Ewertz M., Lorenzen E.L., Brink C. (2018). Breast Cancer Patients Report Reduced Sensitivity and Pain Using a Barrier Film during Radiotherapy—A Danish Intra-Patient Randomized Multicentre Study. Tech. Innov. Patient Support Radiat. Oncol..

[20] Herst P.M., Bennett N.C., Sutherland A.E., Peszynski R.I., Paterson D.B., Jasperse M.L. (2014). Prophylactic Use of Mepitel Film Prevents Radiation-Induced Moist Desquamation in an Intra-Patient Randomised Controlled Clinical Trial of 78 Breast Cancer Patients. Radiother. Oncol..

[21] Rades D., Narvaez C.A., Splettstößer L., Dömer C., Setter C., Idel C., Ribbat-Idel J., Perner S., Bartscht T., Olbrich D. (2019). A Randomized Trial (RAREST-01) Comparing Mepitel^®^ Film and Standard Care for Prevention of Radiation Dermatitis in Patients Irradiated for Locally Advanced Squamous Cell Carcinoma of the Head-and-Neck (SCCHN). Radiother. Oncol..

[22] Yan J., Yuan L., Wang J., Li S., Yao M., Wang K., Herst P.M. (2020). Mepitel Film Is Superior to Biafine Cream in Managing Acute Radiation-Induced Skin Reactions in Head and Neck Cancer Patients: A Randomised Intra-Patient Controlled Clinical Trial. J. Med. Radiat. Sci..

[23] Wooding H., Yan J., Yuan L., Chyou T.-Y., Gao S., Ward I., Herst P.M. (2018). The Effect of Mepitel Film on Acute Radiation-Induced Skin Reactions in Head and Neck Cancer Patients: A Feasibility Study. Br. J. Radiol..

[24] Yee C., Lam E., Gallant F., Karam I., Czarnota G., Soliman H., Wong G., Drost L., Vesprini D., Rakovitch E. (2021). A Feasibility Study of Mepitel Film for the Prevention of Breast Radiation Dermatitis in a Canadian Center. Pract. Radiat. Oncol..

[25] Oshin F., McBrayne L., Bratt M., Lucier M., McKenzie A., Vasiliadis S., Agapito C., D’Alimonte L. (2020). A Retrospective Chart Review on the Prophylactic Use of Mepitel Film for Breast Cancer Patients Undergoing Chest Wall Irradiation: A Single-Institution Experience. J. Med. Imaging Radiat. Sci..

[26] Scott A. (2014). Polymeric Membrane Dressings for Radiotherapy-Induced Skin Damage. Br. J. Nurs..

[27] Hegarty F., Wong M. (2014). Polymeric Membrane Dressing for Radiotherapy-Induced Skin Reactions. Br. J. Nurs..

[28] Schmeel L.C., Koch D., Stumpf S., Leitzen C., Simon B., Schüller H., Vornholt S., Schoroth F., Müdder T., Röhner F. (2018). Prophylactically Applied Hydrofilm Polyurethane Film Dressings Reduce Radiation Dermatitis in Adjuvant Radiation Therapy of Breast Cancer Patients. Acta Oncol..

[29] Schmeel L.C., Koch D., Schmeel F.C., Bücheler B., Leitzen C., Mahlmann B., Kunze D., Heimann M., Brüser D., Abramian A.-V. (2019). Hydrofilm Polyurethane Films Reduce Radiation Dermatitis Severity in Hypofractionated Whole-Breast Irradiation: An Objective, Intra-Patient Randomized Dual-Center Assessment. Polymers.

[30] Arimura T., Ogino T., Yoshiura T., Toi Y., Kawabata M., Chuman I., Wada K., Kondo N., Nagayama S., Hishikawa Y. (2016). Effect of Film Dressing on Acute Radiation Dermatitis Secondary to Proton Beam Therapy. Int. J. Radiat. Oncol. Biol. Phys..

[31] Zou M.Y., Xu D.J., Zhang R., Wang Y.X., Li L., Huang J.T., Ji X.Y. (2021). Study on Prevention of Acute Radiodermatitis with Soft Silicone Film Dressing. Indian J. Pharm. Sci..

[32] White R. (2005). Evidence for Atraumatic Soft Silicone Dressing Use. Wounds.

[33] Zhong W.-H., Tang Q.-F., Hu L.-Y., Feng H.-X. (2013). Mepilex Lite Dressings for Managing Acute Radiation Dermatitis in Nasopharyngeal Carcinoma Patients: A Systematic Controlled Clinical Trial. Med. Oncol..

[34] Diggelmann K.V., Zytkovicz A.E., Tuaine J.M., Bennett N.C., Kelly L.E., Herst P.M. (2010). Mepilex Lite Dressings for the Management of Radiation-Induced Erythema: A Systematic Inpatient Controlled Clinical Trial. Br. J. Radiol..

[35] Paterson D., Poonam P., Bennett N.C., Peszynski R., Beekhuizen M., Herst P. (2012). Randomized Intra-Patient Controlled Trial of Mepilex Lite Dressings versus Aqueous Cream in Managing Radiation-Induced Skin Reactions Post-Mastectomy. J. Cancer Sci. Ther..

[36] Gollins S., Gaffney C., Slade S., Swindell R. (2008). RCT on Gentian Violet versus a Hydrogel Dressing for Radiotherapy-Induced Moist Skin Desquamation. J. Wound Care.

[37] Macmillan M.S., Wells M., MacBride S., Raab G.M., Munro A., MacDougall H. (2007). Randomized Comparison of Dry Dressings versus Hydrogel in Management of Radiation-Induced Moist Desquamation. Int. J. Radiat. Oncol. Biol. Phys..

[38] Bazire L., Fromantin I., Diallo A., de la Lande B., Pernin V., Dendale R., Fourquet A., Savignoni A., Kirova Y.M. (2015). Hydrosorb^®^ versus Control (Water Based Spray) in the Management of Radio-Induced Skin Toxicity: Results of Multicentre Controlled Randomized Trial. Radiother. Oncol..

[39] Ahn S., Sung K., Kim H.J., Choi Y.E., Lee Y.K., Kim J.S., Lee S.K., Roh J.-Y. (2020). Reducing Radiation Dermatitis Using a Film-Forming Silicone Gel during Breast Radiotherapy: A Pilot Randomized-Controlled Trial. In Vivo.

[40] Chan R.J., Blades R., Jones L., Downer T.-R., Peet S.C., Button E., Wyld D., McPhail S., Doolan M., Yates P. (2019). A Single-Blind, Randomised Controlled Trial of StrataXRT^®^—A Silicone-Based Film-Forming Gel Dressing for Prophylaxis and Management of Radiation Dermatitis in Patients with Head and Neck Cancer. Radiother. Oncol..

[41] EP-1286 StrataXRT Is Non Inferior to Mepitel Film in Preventing Radiation Induced Moist Desquamation|Request PDF. https://www.researchgate.net/publication/333212705_EP-1286_StrataXRT_is_non_inferior_to_Mepitel_Film_in_preventing_radiation_induced_moist_desquamation.

[42] Quilis A., Martín J., Rodríguez C., Sánchez P., Ribes J.L. (2018). Reducing Radiation Dermatitis during Ongoing Radiation Therapy: An Innovative Film-Forming Wound Dressing. J. Radiat. Oncol..

[43] Graham P., Browne L., Capp A., Fox C., Graham J., Hollis J., Nasser E. (2004). Randomized, Paired Comparison of No-Sting Barrier Film versus Sorbolene Cream (10% Glycerine) Skin Care during Postmastectomy Irradiation. Int. J. Radiat. Oncol. Biol. Phys..

[44] Lam A.C., Yu E., Vanwynsberghe D., O’Neil M., D’Souza D., Cao J., Lock M. (2019). Phase III Randomized Pair Comparison of a Barrier Film vs. Standard Skin Care in Preventing Radiation Dermatitis in Post-Lumpectomy Patients with Breast Cancer Receiving Adjuvant Radiation Therapy. Cureus.

[45] Shaw S.-Z., Nien H.-H., Wu C.-J., Lui L.T., Su J.-F., Lang C.-H. (2015). 3M Cavilon No-Sting Barrier Film or Topical Corticosteroid (Mometasone Furoate) for Protection against Radiation Dermatitis: A Clinical Trial. J. Formos. Med. Assoc..

[46] Yang X., Ren H., Guo X., Hu C., Fu J. (2020). Radiation-Induced Skin Injury: Pathogenesis, Treatment, and Management. Aging.

[47] Aquino-Parsons C., Lomas S., Smith K., Hayes J., Lew S., Bates A.T., Macdonald A.G. (2010). Phase III Study of Silver Leaf Nylon Dressing vs Standard Care for Reduction of Inframammary Moist Desquamation in Patients Undergoing Adjuvant Whole Breast Radiation Therapy. J. Med. Imaging Radiat. Sci..

[48] Niazi T.M., Vuong T., Azoulay L., Marijnen C., Bujko K., Nasr E., Lambert C., Duclos M., Faria S., David M. (2012). Silver Clear Nylon Dressing Is Effective in Preventing Radiation-Induced Dermatitis in Patients with Lower Gastrointestinal Cancer: Results from a Phase III Study. Int. J. Radiat. Oncol. Biol. Phys..

[49] Vuong T., Franco E., Lehnert S., Lambert C., Portelance L., Nasr E., Faria S., Hay J., Larsson S., Shenouda G. (2004). Silver Leaf Nylon Dressing to Prevent Radiation Dermatitis in Patients Undergoing Chemotherapy and External Beam Radiotherapy to the Perineum. Int. J. Radiat. Oncol. Biol. Phys..

[50] Vavassis P., Gelinas M., Chabot Tr J., Nguyen-Tân P.F. (2008). Phase 2 Study of Silver Leaf Dressing for Treatment of Radiation-Induced Dermatitis in Patients Receiving Radiotherapy to the Head and Neck. J. Otolaryngol. Head Neck Surg..

[51] Beele H. (2002). Artificial Skin: Past, Present and Future. Int. J. Artif. Organs.

[52] Lee J., Lee S., Hong J.P., Shon M.W., Ryu S., Ahn S.D. (2014). Foam Dressing with Epidermal Growth Factor for Severe Radiation Dermatitis in Head and Neck Cancer Patients. Int. Wound J..

[53] Kouvaris J.R., Kouloulias V.E., Plataniotis G.A., Balafouta E.J., Vlahos L.J. (2001). Dermatitis during Radiation for Vulvar Carcinoma: Prevention and Treatment with Granulocyte-Macrophage Colony-Stimulating Factor Impregnated Gauze. Wound Repair Regen..

[54] Lobo Gajiwala A., Sharma V. (2003). Use of Irradiated Amnion as a Biological Dressing in the Treatment of Radiation Induced Ulcers. Cell Tissue Bank..

[55] Bonomo P., Desideri I., Loi M., Ciccone L.P., Lo Russo M., Becherini C., Greto D., Simontacchi G., Pimpinelli N., Livi L. (2019). Management of Severe Bio-Radiation Dermatitis Induced by Radiotherapy and Cetuximab in Patients with Head and Neck Cancer: Emphasizing the Role of Calcium Alginate Dressings. Support. Care Cancer.

[56] Mak S.S., Zee C.Y., Molassiotis A., Chan S.J., Leung S.F., Mo K.F., Johnson P.J. (2005). A Comparison of Wound Treatments in Nasopharyngeal Cancer Patients Receiving Radiation Therapy. Cancer Nurs..

[57] Regulski M.J., Danilkovitch A., Saunders M.C. (2019). Management of a Chronic Radiation Necrosis Wound with Lyopreserved Placental Membrane Containing Viable Cells. Clin. Case Rep..

[58] Wollina U., Liebold K., Konrad H. (2001). Treatment of Chronic Radiation Ulcers with Recombinant Platelet-Derived Growth Factor and a Hydrophilic Copolymer Membrane. J. Eur. Acad. Dermatol. Venereol..

[59] Rigotti G., Marchi A., Galiè M., Baroni G., Benati D., Krampera M., Pasini A., Sbarbati A. (2007). Clinical Treatment of Radiotherapy Tissue Damage by Lipoaspirate Transplant: A Healing Process Mediated by Adipose-Derived Adult Stem Cells. Plast. Reconstr. Surg..

[60] Hom D.B., Manivel J.C. (2003). Promoting Healing with Recombinant Human Platelet-Derived Growth Factor--BB in a Previously Irradiated Problem Wound. Laryngoscope.

[61] Pavel T.I., Chircov C., Rădulescu M., Grumezescu A.M. (2020). Regenerative Wound Dressings for Skin Cancer. Cancers.

[62] Liang Y., He J., Guo B. (2021). Functional Hydrogels as Wound Dressing to Enhance Wound Healing. ACS Nano.

[63] Maleki A., He J., Bochani S., Nosrati V., Shahbazi M.-A., Guo B. (2021). Multifunctional Photoactive Hydrogels for Wound Healing Acceleration. ACS Nano.

[64] Shukla R., Kashaw S.K., Jain A.P., Lodhi S. (2016). Fabrication of Apigenin Loaded Gellan Gum-Chitosan Hydrogels (GGCH-HGs) for Effective Diabetic Wound Healing. Int. J. Biol. Macromol..

[65] Vedakumari W.S., Ayaz N., Karthick A.S., Senthil R., Sastry T.P. (2017). Quercetin Impregnated Chitosan-Fibrin Composite Scaffolds as Potential Wound Dressing Materials—Fabrication, Characterization and in Vivo Analysis. Eur. J. Pharm. Sci..

[66] George D., Maheswari P.U., Begum K.M.M.S. (2019). Synergic Formulation of Onion Peel Quercetin Loaded Chitosan-Cellulose Hydrogel with Green Zinc Oxide Nanoparticles towards Controlled Release, Biocompatibility, Antimicrobial and Anticancer Activity. Int. J. Biol. Macromol..

[67] Jangde R., Srivastava S., Singh M.R., Singh D. (2018). In Vitro and In Vivo Characterization of Quercetin Loaded Multiphase Hydrogel for Wound Healing Application. Int. J. Biol. Macromol..

[68] Ajmal G., Bonde G.V., Thokala S., Mittal P., Khan G., Singh J., Pandey V.K., Mishra B. (2019). Ciprofloxacin HCl and Quercetin Functionalized Electrospun Nanofiber Membrane: Fabrication and Its Evaluation in Full Thickness Wound Healing. Artif. Cells Nanomed. Biotechnol..

[69] Dhivya S., Padma V.V., Santhini E. (2015). Wound Dressings—A Review. Biomedicine.

[70] Derakhshandeh H., Kashaf S.S., Aghabaglou F., Ghanavati I.O., Tamayol A. (2018). Smart Bandages: The Future of Wound Care. Trends Biotechnol..

[71] Konop M., Rybka M., Drapała A. (2021). Keratin Biomaterials in Skin Wound Healing, an Old Player in Modern Medicine: A Mini Review. Pharmaceutics.

[72] Konop M., Czuwara J., Kłodzińska E., Laskowska A.K., Sulejczak D., Damps T., Zielenkiewicz U., Brzozowska I., Sureda A., Kowalkowski T. (2020). Evaluation of Keratin Biomaterial Containing Silver Nanoparticles as a Potential Wound Dressing in Full-Thickness Skin Wound Model in Diabetic Mice. J. Tissue Eng. Regen. Med..

[73] Borek C. (2004). Antioxidants and Radiation Therapy. J. Nutr..

[74] Chao M., Spencer S., Kai C., Baker C., Jassal S., Law M., Cheng M., Zantuck N., Yu V., Stoney D. (2019). EP-1286 StrataXRT Is Non Inferior to Mepitel Film in Preventing Radiation Induced Moist Desquamation. Radiother. Oncol..

