# Supplementary material for: Modern Dressings in Prevention and Therapy of Acute and Chronic Radiation Dermatitis—A Literature Review
Source: Pharmaceutics. 2022 Jun 6;14(6):1204. doi: 10.3390/pharmaceutics14061204 (PMC9229675; doi:10.3390/pharmaceutics14061204)
Supplement: Supplementary file 1 [file pharmaceutics-14-01204-s001.zip › pharmaceutics-1684598-supplementary.pdf]

Table S1. Proposed T.I.M.E. wound bed preparation for acute and chronic radiation dermatitis

Type of radiation dermatits	T.I.M.E. domain	Actions
Acute	T – tissue management	Debridement during radiotherapy is rarely performed in the clinical practice due to the possible impact on oncological treatment (dose distribution, a risk of seeding of cancer cells).
	I – inflammation and infection control	Moderate and severe acute radiation dermatitis may be related to secondary infections due to the immunosuppression, skin damage and concomitant systemic treatment. In the case of mild infections topical therapies with silver-coated dressings or topical antimicrobials should be considered. Skin infections with general symptoms (severe edema, fever, pain) are the indications for oral antibiotics.
	M – moisture balance	The important part of the prevention and treatment of acute radiation dermatitis is the moisture balance. Severe acute radiation dermatitis is related to moist desquamation. Thus, appropriate hydration and protection of the skin using paraffin-coated dressing or radiotherapy-dedicated creams is recommended. In the case of severe exudate, film and hydrocolloid dressings should be avoided.
	E - epithelial edge advancement	It is important to eliminate or reduce extrinsic factors that may impair the reepithelization such as too tight clothes, repeated trauma, or the use of other irritating factors (such as chlorine water, antiperspirants).
Chronic	T – tissue management	Appropriate types of debridement (surgical, enzymatic) in the case of necrosis should be considered after the exclusion of cancer recurrence (local regrowth may mimic chronic radiation dermatitis) or secondary cancer.
	I – inflammation and infection control	Rules are similar to those in other infected wounds.
	M – moisture balance	Wounds related to chronic radiation dermatitis are rarely exudative. Atrophic changes require intensive moisturizing (creams for atopic dermatitis).
	E - epithelial edge advancement	Hyperbaric oxygen therapy may be used in selected patients to improve healing. A special attention should be paid on the possibility of ischemia with chronic hypoxia after high-dose radiotherapy that causes irreversible endothelial damage.

Table S2. Dressings for prevention and treatment of acute radiation dermatitis

**Dressing classification**	**Dressing type**	**Study**	**Study type**	**Sample size**	**Cancer type**	**Aim**	**Clinical outcome**	**Confirmed efficacy (met endpoint)**
Film or membrane	Mepitel film	Herst et al., 2014 [1]	RCT	78	breast	ARD prophylaxis	complete prevention of moist desquamation, 92% reduction of skin reaction severity	yes
		Rades et al., 2019 [2]	RCT	28	head and neck	ARD prophylaxis	not superior to standard care, prematurely stopped (tolerance)	no
		Wooding et al., 2018 [3]	RCT	36	head and neck	ARD prophylaxis	reduced risk of developing ARD	yes
		Yee et al., 2020 [4]	single arm CT	30	breast, chest wall	ARD prophylaxis	prevention of grade 3 ARD, reduction of grade 2 ARD cases, feasibility confirmed	yes
		Møller et al., 2018 [5]	RCT	101	breast	ARD prophylaxis	reduction of patient-reported symptoms, lower severity of ARD, no difference in the blinded-staff evaluation at follow-up	yes (primary endpoint)
		Yan et al., 2020 [6]	RCT	39	head and neck	ARD prophylaxis	reduces severity of ARD including moist desquamation compared to Biafine cream	yes

		Oshin et al., 2020 [7]	retrospective review	15	chest wall	ARD prophylaxis	reduction of moist desquamation incidence and severity	yes
	Soft silicone film dressing	Zou et al., 2021 [8]	RCT	100	various	ARD prophylaxis	lower incidence of ARD in experimental arm	yes
	StrataXRT®	Chan et al., 2019 [9]	RCT	197	head and neck	ARD prophylaxis	reduced risk of developing grade 2 and 3 skin toxicity	yes
		Ahn et al., 2020 [10]	RCT	56	breast	ARD prophylaxis	reduced objectively measured ARD severity	yes
		Quilis et al., 2018 [11]	multicenter prospective CT	54	various	ARD treatment	significant improvement in RISRAS score, reduction of patient-reported symptoms	yes

		Chao et al., 2019 [12]	noninferiority RCT	44	breast	ARD prophylaxis	StrataXRT not inferior to Mepitel FIlm	yes
	Polymeric membrane dressing	Scott, 2014 [13]	RCT	20	head and neck	ARD treatment	reduced self-reported pain, improved quality of life, no effect on healing rates	mixed
		Hegarty et al., 2014 [14]	single arm CT	23	head and neck	ARD treatment	superior to standard care	yes
	Hydrofilm (polyurethane film)	Schmeel et al., 2018 [15]	RCT	62	breast	ARD prophylaxis	ARD severity reduction, preventing of moist desquamation	yes
		Schmeel et al., 2019 [16]	self-controlled trial	74	breast	ARD prophylaxis	reduction of physician-assessed ARD, decreased erythema and hyperpigmentation, prevention of moist desquamation, reduction of itching, burning, pain, and limitations of day-to-day-activities	yes

Silver	Silver nylon dressings	Aquino-Parsons et al., 2010 [17]	RCT	196	breast	ARD prophylaxis	no decrease in the incidence of moist desquamation, decrease in itching	no (only secondary endpoint was met)
		Vuong et al., 2004 [18]	single arm CT	30	gynaecological, anal	ARD prophylaxis	effective in reducing RID severity (compared to historical controls)	yes
		Vavassis et al., 2008 [19]	self-controlled trial	12	head and neck	ARD treatment	not superior to standard care	no
		Niazi et al., 2012 [20]	RCT	42	anal and rectal cancer	ARD prophylaxis	effective in reducing RID severity on the last day of treatment but not 2 weeks post-treatment	yes
Foam	Mepilex Lite	Diggelmann et al., 2010 [21]	self-controlled trial	24	breast	ARD treatment	reduction of radiation-induced erythema	yes
		Zhong et al., 2013 [22]	self-controlled trial	88	head and neck	ARD treatment	acceleration of ARD healing	yes
		Paterson et al., 2012 [23]	self-controlled trial	74	breast	ARD treatment	decreases the severity of all skin reactions	yes
Gel	Hydrogel	Gollins et al., 2008 [24]	RCT	30	head and neck, breast	ARD treatment	hydrogel dressings superior to gentian violet in increasing healing of moist desquamation	yes
		Macmillan et al., 2007 [25]	RCT	100	head and neck, breast, anorectal	ARD treatment	prolonged healing times in hydrogel group	no
		Bazire et al., 2015 [26]	RCT	186	breast	ARD treatment	no difference between Hydrosorb and control groups	no
	3M Cavilon Barrier Film	Lam et al., 2019 [27]	RCT	55	breast	ARD prophylaxis	no significant reduction of ARD during or post-treatment (paired analysis), significant reduction of ARD in lateral compartment (unpaired analysis) during treatment but not post-treatment, reduction of pruritus and burning sensations.	no

		Graham et al., 2004 [28]	RCT	61	breast	ARD prophylaxis	reduction (vs sorbolene cream) of moist desquamation rates and pruritus	yes
		Shaw et al., 2015 [29]	RCT	39	breast	ARD prophylaxis	no significant differences in pain, pruritus and ARD severity after application of the film	no
Biodressing	GM-CSF-impregnated gauze	Kouvaris et al., 2001 [30]	retrospective cohort analysis	61	vulvar carcinoma	ARD prophylaxis and treatment	effective in preventing and healing radiation-induced dermatitis	yes
	Foam dressing with EGF	Lee et al., 2016 [31]	single arm CT	7	head and neck	ARD treatment	acceleration of healing of severe ARD on the supraclavicular fossa or neck areas	yes
	Irradiated, lyophilised amnion	Lobo Gajiwala et al., 2003 [32]	single-arm CT	14	cervix, rectum	ARD treatment	shorter duration of healing (moist desquamation/ulcers)	yes
Other	Airwall	Arimura et al., 2016 [33]	non-randomized CT	271	prostate	ARD prophylaxis (proton therapy)	ARD severity reduction	yes
	Calcium alginate	Bonomo et al., 2019 [34]	retrospective cohort analysis	51	head and neck	ARD treatment (radiotherapy with cetuximab)	improval of treatment tolerability	yes
	Nonadherent absorbent dressing	Mak et al., 2005 [35]	RCT	146	head and neck	ARD treatment	no difference between dressing and gentian violet groups in reduction of moist desquamation	no

Abbreviations: ARD - acure radiation dermatitis, CT - clinical trial, EGF - epidermal growth factor, GM-CSF - granulocyte-macrophage colony-stimulating factor, RCT - randomized clinical trial, RISRAS - Radiation-induced Skin Reaction Assessment Scale

References

1. Herst PM, Bennett NC, Sutherland AE, Peszynski RI, Paterson DB, Jasperse ML. Prophylactic use of Mepitel Film prevents radiation-induced moist desquamation in an intra-patient randomised controlled clinical trial of 78 breast cancer patients. *Radiother Oncol*. 2014;110(1):137-143. doi:10.1016/j.radonc.2014.01.005
2. Rades D, Narvaez CA, Splettstößer L, et al. A randomized trial (RAREST-01) comparing Mepitel® Film and standard care for prevention of radiation dermatitis in patients irradiated for locally advanced squamous cell carcinoma of the head-and-neck (SCCHN). *Radiother Oncol*. 2019;139:79-82. doi:10.1016/j.radonc.2019.07.023
3. Wooding H, Yan J, Yuan L, et al. The effect of Mepitel Film on acute radiation-induced skin reactions in head and neck cancer patients: a feasibility study. *Br J Radiol*. 2018;91(1081):20170298. doi:10.1259/bjr.20170298
4. Yee C, Lam E, Gallant F, et al. A Feasibility Study of Mepitel Film for the Prevention of Breast Radiation Dermatitis in a Canadian Center. *Pract Radiat Oncol*. 2021;11(1):e36-e45. doi:10.1016/j.prro.2020.09.004
5. Møller PK, Olling K, Berg M, et al. Breast cancer patients report reduced sensitivity and pain using a barrier film during radiotherapy – A Danish intra-patient randomized multicentre study. *Tech Innov Patient Support Radiat Oncol*. 2018;7:20-25. doi:10.1016/j.tipsro.2018.05.004
6. Yan J, Yuan L, Wang J, et al. Mepitel Film is superior to Biafine cream in managing acute radiation-induced skin reactions in head and neck cancer patients: a randomised intra-patient controlled clinical trial. *J Med Radiat Sci*. 2020;67(3):208-216. doi:10.1002/jmrs.397
7. Oshin F, McBrayne L, Bratt M, et al. A Retrospective Chart Review on the Prophylactic Use of Mepitel Film for Breast Cancer Patients undergoing Chest Wall Irradiation: A Single-institution Experience. *J Med Imaging Radiat Sci*. 2020;51(3):S3-S4. doi:10.1016/j.jmir.2020.07.014
8. Zou MY, Xu DJ, Zhang R, et al. Study on Prevention of Acute Radiodermatitis with Soft Silicone Film Dressing. *Indian J Pharm Sci*. 2021;83. doi:10.36468/pharmaceutical-sciences.spl.250
9. Chan RJ, Blades R, Jones L, et al. A single-blind, randomised controlled trial of StrataXRT® – A silicone-based film-forming gel dressing for prophylaxis and management of radiation dermatitis in patients with head and neck cancer. *Radiother Oncol*. 2019;139:72-78. doi:10.1016/j.radonc.2019.07.014
10. Ahn S, Sung K, Kim HJ, et al. Reducing Radiation Dermatitis Using a Film-forming Silicone Gel During Breast Radiotherapy: A Pilot Randomized-controlled Trial. *In Vivo*. 2020;34(1):413-422. doi:10.21873/invivo.11790
11. Quilis A, Martín J, Rodríguez C, Sánchez P, Ribes JL. Reducing radiation dermatitis during ongoing radiation therapy: an innovative film-forming wound dressing. *J Radiat Oncol*. 2018;7(3):255-264. doi:10.1007/s13566-018-0356-5
12. Chao MWT, Spencer S, Kai C, et al. EP-1286 StrataXRT is non inferior to Mepitel Film in preventing radiation induced moist desquamation. *Radiother Oncol*. 2019;133:S704-S705. doi:10.1016/S0167-8140(19)31706-2
13. Scott A. Polymeric membrane dressings for radiotherapy-induced skin damage. *Br J Nurs*. 2014;23(Sup10):S24-S31. doi:10.12968/bjon.2014.23.Sup10.S24
14. Hegarty F, Wong M. Polymeric membrane dressing for radiotherapy-induced skin reactions. *Br J Nurs*. 2014;23(Sup20):S38-S46. doi:10.12968/bjon.2014.23.Sup20.S38
15. Schmeel LC, Koch D, Stumpf S, et al. Prophylactically applied Hydrofilm polyurethane film dressings reduce radiation dermatitis in adjuvant radiation therapy of breast cancer patients. *Acta Oncol*. 2018;57(7):908-915. doi:10.1080/0284186X.2018.1441542
16. Schmeel LC, Koch D, Schmeel FC, et al. Hydrofilm Polyurethane Films Reduce Radiation Dermatitis Severity in Hypofractionated Whole-Breast

Irradiation: An Objective, Intra-Patient Randomized Dual-Center Assessment. *Polymers*. 2019;11(12):E2112. doi:10.3390/polym11122112
17. Aquino-Parsons C, Lomas S, Smith K, et al. Phase III Study of Silver Leaf Nylon Dressing vs Standard Care for Reduction of Inframammary Moist Desquamation in Patients Undergoing Adjuvant Whole Breast Radiation Therapy. *J Med Imaging Radiat Sci*. 2010;41(4):215-221. doi:10.1016/j.jmir.2010.08.005
18. Vuong T, Franco E, Lehnert S, et al. Silver leaf nylon dressing to prevent radiation dermatitis in patients undergoing chemotherapy and external beam radiotherapy to the perineum. *Int J Radiat Oncol*. 2004;59(3):809-814. doi:10.1016/j.ijrobp.2003.11.031
19. Vavassis P, Gelinas M, Chabot Tr J, Nguyen-Tân PF. Phase 2 study of silver leaf dressing for treatment of radiation-induced dermatitis in patients receiving radiotherapy to the head and neck. *J Otolaryngol - Head Neck Surg J Oto-Rhino-Laryngol Chir Cervico-Faciale*. 2008;37(1):124-129.
20. Niazi TM, Vuong T, Azoulay L, et al. Silver Clear Nylon Dressing is Effective in Preventing Radiation-Induced Dermatitis in Patients With Lower Gastrointestinal Cancer: Results From a Phase III Study. *Int J Radiat Oncol*. 2012;84(3):e305-e310. doi:10.1016/j.ijrobp.2012.03.062
21. Diggelmann KV, Zytkovicz AE, Tuaine JM, Bennett NC, Kelly LE, Herst PM. Mepilex Lite dressings for the management of radiation-induced erythema: a systematic inpatient controlled clinical trial. *Br J Radiol*. 2010;83(995):971-978. doi:10.1259/bjr/62011713
22. Zhong WH, Tang QF, Hu LY, Feng HX. Mepilex Lite dressings for managing acute radiation dermatitis in nasopharyngeal carcinoma patients: a systematic controlled clinical trial. *Med Oncol*. 2013;30(4):761. doi:10.1007/s12032-013-0761-y
23. Paterson D. Randomized Intra-patient Controlled Trial of Mepilex Lite Dressings versus Aqueous Cream in Managing Radiation-Induced Skin Reactions Postmastectomy. *J Cancer Sci Ther*. 2012;04(11). doi:10.4172/1948-5956.1000166
24. Gollins S, Gaffney C, Slade S, Swindell R. RCT on gentian violet versus a hydrogel dressing for radiotherapy-induced moist skin desquamation. *J Wound Care*. 2008;17(6):268-275. doi:10.12968/jowc.2008.17.6.29589
25. Macmillan MS, Wells M, MacBride S, Raab GM, Munro A, MacDougall H. Randomized Comparison of Dry Dressings Versus Hydrogel in Management of Radiation-Induced Moist Desquamation. *Int J Radiat Oncol*. 2007;68(3):864-872. doi:10.1016/j.ijrobp.2006.12.049
26. Bazire L, Fromantin I, Diallo A, et al. Hydrosorb® versus control (water based spray) in the management of radio-induced skin toxicity: Results of multicentre controlled randomized trial. *Radiother Oncol*. 2015;117(2):229-233. doi:10.1016/j.radonc.2015.08.028
27. Lam AC, Yu E, Vanwynsberghe D, et al. Phase III Randomized Pair Comparison of a Barrier Film vs. Standard Skin Care in Preventing Radiation Dermatitis in Post-lumpectomy Patients with Breast Cancer Receiving Adjuvant Radiation Therapy. *Cureus*. Published online June 3, 2019. doi:10.7759/cureus.4807
28. Graham P, Browne L, Capp A, et al. Randomized, paired comparison of No-Sting Barrier Film versus sorbolene cream (10% glycerine) skin care during postmastectomy irradiation. *Int J Radiat Oncol*. 2004;58(1):241-246. doi:10.1016/S0360-3016(03)01431-7
29. Shaw SZ, Nien HH, Wu CJ, Lui LT, Su JF, Lang CH. 3M Cavilon No-Sting Barrier Film or topical corticosteroid (mometasone furoate) for protection against radiation dermatitis: A clinical trial. *J Formos Med Assoc*. 2015;114(5):407-414. doi:10.1016/j.jfma.2013.04.003
30. Kouvaris JR, Kouloulias VE, Plataniotis GA, Balafouta EJ, Vlahos LJ. Dermatitis during radiation for vulvar carcinoma: prevention and treatment with granulocyte-macrophage colony-stimulating factor impregnated gauze. *Wound Repair Regen*. 2001;9(3):187-193. doi:10.1046/j.1524-475x.2001.00187.x
31. Lee J, Lee S wook, Hong JP, Shon MW, Ryu SH, Ahn SD. Foam dressing with epidermal growth factor for severe radiation dermatitis in head and neck cancer patients: Foam dressing with EGF for radiation dermatitis. *Int Wound J*. 2016;13(3):390-393. doi:10.1111/iwj.12317
32. Lobo Gajiwala A, Sharma V. Use of Irradiated Amnion as a Biological Dressing in the Treatment of Radiation Induced Ulcers. *Cell Tissue Bank*.

2003;4(2-4):147-150. doi:10.1023/B:CATB.0000007024.81019.03
33. Arimura T, Ogino T, Yoshiura T, et al. Effect of Film Dressing on Acute Radiation Dermatitis Secondary to Proton Beam Therapy. *Int J Radiat Oncol*. 2016;95(1):472-476. doi:10.1016/j.ijrobp.2015.10.053
34. Bonomo P, Desideri I, Loi M, et al. Management of severe bio-radiation dermatitis induced by radiotherapy and cetuximab in patients with head and neck cancer: emphasizing the role of calcium alginate dressings. *Support Care Cancer*. 2019;27(8):2957-2967. doi:10.1007/s00520-018-4606-2
35. Mak S, Zee C, Molassiotis A, et al. A Comparison of Wound Treatments in Nasopharyngeal Cancer Patients Receiving Radiation Therapy: *Cancer Nurs*. 2005;28(6):436-545. doi:10.1097/00002820-200511000-00005
